# Human umbilical cord-derived mesenchymal stem cell therapy in patients with COVID-19: a phase 1 clinical trial

**DOI:** 10.1038/s41392-020-00286-5

**Published:** 2020-08-27

**Authors:** Fanping Meng, Ruonan Xu, Siyu Wang, Zhe Xu, Chao Zhang, Yuanyuan Li, Tao Yang, Lei Shi, Junliang Fu, Tianjun Jiang, Lei Huang, Peng Zhao, Xin Yuan, Xing Fan, Ji-Yuan Zhang, Jinwen Song, Dawei Zhang, Yanmei Jiao, Limin Liu, Chunbao Zhou, Markus Maeurer, Alimuddin Zumla, Ming Shi, Fu-Sheng Wang

**Affiliations:** 1 Department of Infectious Diseases, Fifth Medical Center of Chinese PLA General Hospital, National Clinical Research Center for Infectious Diseases, Beijing, China; 2 Immunotherapy Programme, Champalimaud Centre for the Unknown, Lisbon, Portugal; 3 I Med Clinic, University of Mainz, Mainz, Germany; 4 Department of Infection, Division of Infection and Immunity, University College London, London, UK; 5 National Institute for Health Research Biomedical Research Centre, University College London Hospitals NHS Foundation Trust, London, UK

**Keywords:** Stem-cell research, Drug development

## Abstract

No effective drug treatments are available for coronavirus disease 2019 (COVID-19). Host-directed therapies targeting the underlying aberrant immune responses leading to pulmonary tissue damage, death, or long-term functional disability in survivors require clinical evaluation. We performed a parallel assigned controlled, non-randomized, phase 1 clinical trial to evaluate the safety of human umbilical cord-derived mesenchymal stem cells (UC-MSCs) infusions in the treatment of patients with moderate and severe COVID-19 pulmonary disease. The study enrolled 18 hospitalized patients with COVID-19 (*n* = 9 for each group). The treatment group received three cycles of intravenous infusion of UC-MSCs (3 × 10^7^ cells per infusion) on days 0, 3, and 6. Both groups received standard COVID-treatment regimens. Adverse events, duration of clinical symptoms, laboratory parameters, length of hospitalization, serial chest computed tomography (CT) images, the PaO_2_/FiO_2_ ratio, dynamics of cytokines, and IgG and IgM anti-SARS-CoV-2 antibodies were analyzed. No serious UC-MSCs infusion-associated adverse events were observed. Two patients receiving UC-MSCs developed transient facial flushing and fever, and one patient developed transient hypoxia at 12 h post UC-MSCs transfusion. Mechanical ventilation was required in one patient in the treatment group compared with four in the control group. All patients recovered and were discharged. Our data show that intravenous UC-MSCs infusion in patients with moderate and severe COVID-19 is safe and well tolerated. Phase 2/3 randomized, controlled, double-blinded trials with long-term follow-up are needed to evaluate the therapeutic use of UC-MSCs to reduce deaths and improve long-term treatment outcomes in patients with serious COVID-19.

## Introduction

Severe acute respiratory syndrome coronavirus-2 (SARS-CoV-2) infection in humans has led to an ongoing pandemic of coronavirus disease 2019 (COVID-19) worldwide. COVID-19 causes a spectrum of clinical illness from mild, moderate, to severe or critical disease.^1^ As of 6 July 2020, there have been 532,340 deaths from COVID-19 out 11,327,790 cases reported to the World Health Organization.^2^ While COVID-19 predominantly affects the respiratory tract, it is a multisystem disease, and SARS-CoV-2 antigens have been detected in most organs. Severe cases of COVID-19 are typically characterized by upregulation of pro-inflammatory cytokines and chemokines, aberrant cellular immune responses, abnormal coagulation indices, respiratory and cardiovascular failure, end organ damage, and even death.^1,3^ It is likely that aberrant and excessive immune responses evoked by SARS-CoV-2 infection in the host are involved in the pathogenesis of lung and multi-organ injury.^4,5^

To date, no specific antivirals have proven to be effective to treat COVID-19 and the management of patients with COVID-19 remains largely supportive, with organ support when necessary. Thus, deaths from COVID-19 will continue to rise globally until new effective therapeutics are developed and evaluated in clinical trials. In addition to anti-SARS-CoV-2 drugs or treatment combinations, other treatment options need to be considered.

Host-directed therapies that target the underlying aberrant immune responses leading to pulmonary tissue damage, death, or long-term consequential functional disability in those who survive, require evaluation in clinical trials.^6^ Mesenchymal stem cells (MSCs) are non-hematopoietic cells that have immune modulatory, regenerative, and differentiation properties.^7,8^ MSCs were first tested as a cellular therapy in humans in 1995 and have since been used in basic research and clinical applications.^9^ MSCs are considered to suppress the over-activated inflammatory response, promote recovery of lung function, and potentially influence the progress of pulmonary fibrosis.^10^ Thus, the use of MSCs to improve the clinical outcome of the patients with severe cases of COVID-19 disease requires evaluation.

MSCs derived from different tissues, including human bone marrow, umbilical cord tissue, adipose tissue, lung tissue, dental pulp, and placenta,^7^ have been used in humans, without serious adverse events to treat corticosteroid-resistant graft-versus-host disease, multiple sclerosis, heart failure, acute respiratory distress syndrome (ARDS), and other indications.^11–14^ However, their use and safety profile in patients with COVID-19 needs to be determined in light of the multi-system nature of disease associated coagulopathy. Therefore, we performed a parallel assigned, controlled, non-randomized, phase 1 clinical trial to evaluate the safety of human umbilical cord-derived mesenchymal stem cells infusions in the treatment of patients with moderate and severe COVID-19 during the early phase of the COVID-19 pandemic since 27 January 2020.

## Results

### Baseline characteristics of the enrolled patients

**Fig. 1:**
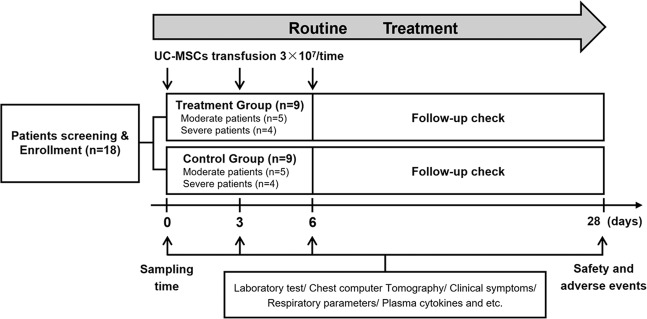
Trial Profile. Structure and patient enrollment of the non-randomized, parallel trial

A total of 18 patients were enrolled, nine of whom (five with moderate disease and four with severe disease) received three cycles of UC-MSCs treatment. The other nine patients (five with moderate disease and four with severe disease) were assigned to the control group (Fig. 1). Baseline characteristics of all patients, including age, sex, clinical classification, co-existing co-morbidities, the interval between symptoms onset and admission, and medical treatment were recorded (Table 1). One patient (C1) in the control group, and two patients (T5 and T9) in the UC-MSCs treatment group were viral RNA negative at enrollment, the others were positive for plasma viral RNA. The duration from first symptom onset to hospital admission in the UC-MSCs treatment group ranged from day 3 to 15; in the control group it ranged from day 4 to 11.

**Table 1:** Baseline characteristics of enrolled patients with COVID-19

	UC-MSCs treatment group (*n* = 9)									Control group (*n* = 9)								
Patient number	T1	T2	T3	T4	T5	T6	T7	T8	T9	C1	C2	C3	C4	C5	C6	C7	C8	C9
Age (years)	45	44	45	39	64	40	36	59	34	54	44	53	67	37	33	48	60	50
Sex	M	M	F	F	M	M	M	M	M	F	F	F	M	M	M	M	F	F
Severity of disease	Mod	Mod	Mod	Mod	Mod	Sev	Sev	Sev	Sev	Mod	Mod	Mod	Mod	Mod	Sev	Sev	Sev	Sev
Co-existing presentation diseases	No	HTN	No	No	No	Diabetes	No	HTN	Fatty liver disease	No	No	No	No	HTN	No	No	Asthma	No
Interval between symptom onset and admission (days)	3	6	6	7	15	3	3	7	10	4	8	8	6	5	7	6	6	11
Viral RNA at enrollment	+	+	+	+	−	+	+	+	−	−	+	+	+	+	+	+	+	+
Antivirals treatment	LPV/r	LPV/r	LPV/r	LPV/r	LPV/r	LPV/r	LPV/r	LPV/r	No	LPV/r	LPV/r	LPV/r	No	LPV/r	LPV/r	LPV/r	LPV/r	LPV/r
Steroids treatment	GC	GC	GC	GC	GC	GC	GC	GC	GC	No	No	No	GC	GC	GC	GC	GC	GC

*T* treatment, *C* control, *M* male, *F* female, *Mod* moderate, *Sev* severe, *HTN* hypertension, *LPV/r* lopinavir/ritonavir, *GC* glucocorticoid

### Adverse events during UC-MSCs treatment

There were no serious adverse events associated with UC-MSCs infusion (Table 2). Two patients receiving UC-MSCs developed transient facial flushing and fever immediately on infusion, which resolved spontaneously within 4 h. Another patient with moderate disease had a transient fever (38 °C) within 2 h that resolved within 24 h. Electrocardiography and pulse oxygen saturation monitoring were conducted during cell transfusion, and no electrocardiography abnormalities occurred in any of the patients, while patient T6 experienced hypoxemia within 12 h after UC-MSCs treatment and recovered within 36 h after receiving humidified high-flow nasal cannula oxygen therapy, which was thought to be caused by the progression of COVID-19 based on previously existing symptoms. The above-mentioned findings indicated that UC-MSCs treatment for patients with COVID-19 was safe and tolerable.

**Table 2:** Side effects in patients receiving UC-MSCs infusions

Patient number	Clinical disease severity	Infusion round	EKG monitoring	POS monitoring (%)	Infusion associated events
Patient T1	Moderate	First infusion	Normal	98	Facial flushing occurred within 4 h after UC-MSCs transfusion and spontaneously relieved within 24 h
Patient T4	Moderate	Third infusion	Normal	100	Transient fever no more than 38 °C within 2 h after UC-MSCs transfusion and spontaneously relieved within 24 h
Patient T6	Severe	First infusion	Normal	91–99	Severe hypoxemia occurred within 12 h after UC-MSCs transfusion, believed to be associated with the progression of COVID-19, and recovered after HFNC (Humidified high-flow nasal cannula oxygen therapy)

*EKG* electrocardiography, *POS* pulse oxygen saturation

### Outcomes of cases with/without UC-MSCs treatment

During the follow-up period, all 18 patients recovered and were discharged from our hospital. The clinical characteristics of the two patient groups at discharge were recorded (Table 3). The duration from admission to discharge in the treated group and control group was same (20.00 vs 23.00 days, *P* = 0.306). During hospitalization, one patient in the UC-MSCs treatment group needed mechanical ventilation for 1 day and one patient experienced shortness of breath. In the control group, four patients needed mechanical ventilation and five patients experienced dyspnea. The anti-SARS-CoV-2 IgM antibody tests were positive for all patients. The median IgG (19.93 vs 21.50 signal-to-cut-off ratio, *P* = 0.174) and IgM (24.62 *vs* 76.89 signal-to-cut-off ratio, *P* = 0.114) antibodies titer numerically but not statistically decreased in the UC-MSCs treatment group compared with that of the control group.

**Table 3:** Comparison of clinical data among the patients with COVID-19 with/without UC-MSCs treatment at discharge

Clinical data	UC-MSCs treatment group (*n* = 9)	Control group (*n* = 9)	*P* value
Mechanical ventilation	1/9	4/9	0.294
Oxygen support (duration days for each case)			
Low-flow oxygen support	7/9	7/9	1.000
High-flow oxygen support	3/9	5/9	0.637
Clinical symptoms (duration days for each case)			
Fever	5/9	2/9	0.335
Fatigue	4/9	5/9	1.000
Cough	4/9	8/9	0.131
Shortness of breath	1/9	5/9	0.131
Antibody at discharge (titer of antibody, s/co)			
IgM antibody	24.62 (8.43, 45.97)	76.89 (19.13, 187.20)	0.114
IgG antibody	19.93 (3.37, 33.59)	21.50 (15.69, 121.10)	0.174
Interval between admission and discharge (days)	20.00 (17.50, 24.50)	23.00 (20.00, 27.00)	0.306

Data are shown as *n*/*N*, and the duration for each case was shown. Titer of IgM, IgG, and interval between admission and discharge were shown as median (IQR)

**Fig. 2:**
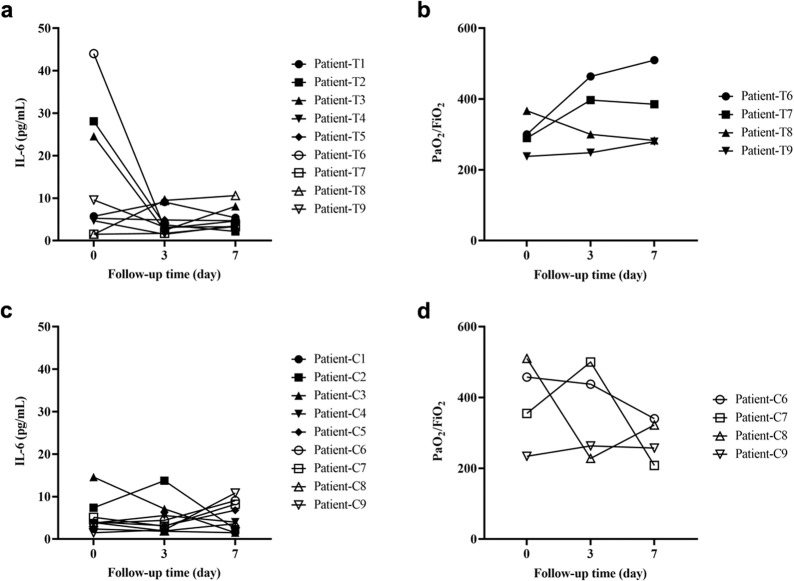
Changes in IL-6 and the PaO_2_/FiO_2_ ratio in patients with COVID-19 with/without UC-MSCs transfusion on day 0, 3, and 7. The changes in IL-6 and the PaO_2_/FiO_2_ ratio were recorded at day 0, 3, and 7 after UC-MSCs treatment. **a** The changes in serum IL-6 levels in the patients receiving UC-MSCs treatment. **b** The changes in the PaO_2_/FiO_2_ ratio in patients with severe disease receiving UC-MSCs treatment. **c** The changes in IL-6 levels in the control group. **d** The changes in the PaO_2_/FiO_2_ ratio in patients with severe disease in the control group

Laboratory test included C-reactive protein (CRP), alanine aminotransferase (ALT), creatinine, serum ferritin (SF), and platelets before and after UC-MSCs treatment at day 0, 3, and 7. As shown in Table 4, the paralleled parameters were also recorded in the control group. The data showed that the laboratory parameters improved in both groups. In the UC-MSCs treatment group, two patients with moderate disease and two with severe disease with high baseline interleukin (IL)-6 exhibited a decline of IL-6 within 3 days after UC-MSCs infusion and remained stable during the following 4 days. We observed no such trend in the patients with lower plasma IL-6 levels, which suggested that the patients with high IL-6 might be more likely benefit from UC-MSCs treatment. In most severe patients, the partial pressure of arterial oxygen:percentage of inspired oxygen (PaO_2_/FiO_2_) ratio improved after UC-MSCs treatment (Fig. 2). CT scans indicated that patients showed absorption of pulmonary pathological changes. Representative chest CT scan images of severe patient 9 after UC-MSCs transfusion showed that the lung lesions were well controlled within 6 days, and completely faded away within 2 weeks after UC-MSCs transfusion. By contrast, the lung lesions of the severe patient 7 in the control group still existed at discharge (Supplementary Fig 1). Interestingly, when the dynamics of a large panel of inflammatory cytokines (including interferon gamma (IFN-γ), tumor necrosis factor alpha (TNF-α), monocyte chemoattractant protein 1 (MCP-1), interferon-inducible cytokine IP-10 (IP-10), IL-22, interleukin 1 receptor type 1 (IL-1RA), IL-18, IL-8, and macrophage inflammatory protein 1-alpha (MIP-1)) were simultaneously monitored in UC-MSCs-treated patients, we found there was a reduced trend in the levels of all these cytokines within 14 days (Supplementary Fig 2).

**Table 4:** Laboratory parameters in the patients with COVID-19 with/without UC-MSCs transfusion on day 0, 3, and 7

	UC-MSCs treatment group (*n* = 9)									Control group (*n* = 9)								
	T1	T2	T3	T4	T5	T6	T7	T8	T9	C1	C2	C3	C4	C5	C6	C7	C8	C9
*CRP, mg/L*																		
Day 0	19.7	6.9	9.3	1.2	2.4	36.4	2.98	1.16	1.62	0.5	8.3	19.73	3.55	5.31	1.39	39.4	2.08	3.65
Day 3	11.80	1.00	18.10	0.24	5.78	14.40	1.70	27.59	1.07	0.22	11	6.78	1.24	14.12	0.43	6	0.72	1.62
Day 7	2.29	3.01	0.09	0.15	3.66	0.62	2.96	30.16	0.77	0.28	2.69	2.93	0.56	7.6	2.73	2.25	0.29	3.84
*Platelets, ×10*^*9*^*/L*																		
Day 0	214	158	290	209	271	134	241	292	212	167	188	201	222	191	420	126	214	355
Day 3	154	297	320	278	284	218	374	192	167	191	191	261	276	250	357	223	246	318
Day 7	226	169	321	368	249	217	348	156	143	143	242	276	236	257	207	202	240	218
*ALT, U/L*																		
Day 0	33	32	22	14	14	33	32	19	71	14	13	12	185	20	27	13	17	76
Day 3	19	17	39	19	16	10	47	21	57	14	9	14	168	28	47	10	21	45
Day 7	32	29	13	31	13	19	70	37	36	14	13	16	75	30	72	49	22	31
*Creatine, μmol/L*																		
Day 0	85	96	70	70	78	82	69	68	63	83	86	56	78	74	67	82	66	64
Day 3	104	60	82	71	77	71	76	65	64	81	73	59	76	70	71	73	60	70
Day 7	77	93	57	65	65	64	72	67	73	81	71	57	78	91	75	73	53	58
*D-dimer, mg/L*																		
Day 0	0.39	0.15	0.57	0.24	0.3	0.13	0.42	0.2	1.29	0.31	0.19	0.33	0.37	0.74	1.12	0.16	0.37	2.31
Day 3	0.18	0.62	0.42	0.20	0.51	0.43	0.31	0.31	1.10	0.26	0.17	0.42	0.61	0.11	1.6	0.23	0.94	1.1
Day 7	0.51	0.19	1.07	0.71	0.55	0.69	0.18	0.4	1.41	0.24	0.18	0.49	033	0.18	4.67	0.97	4.41	0.93
*SF, ng/mL*																		
Day 0	228.6	63.3	1090	150.1	139.5	323.3	386.6	475.6	1148	132.7	212.8	265.2	709.8	633.1	1139	408.7	311.4	818.3
Day 3	336	54	823	332	155	434	568	540	1028	147.3	228.8	280.4	625	556.4	1021	519	256.6	770.5
Day 7	416.6	91.9	798.3	295.8	130.8	785.9	689.6	549.9	763.2	125.5	244.5	292.6	449.9	191	1649	528.7	500.1	1166

*CRP* C-reactive protein, *ALT* alanine aminotransferase, *SF* serum ferritin

## Discussion

COVID-19 is a multisystem disease that is, at least in part, similar to previous findings in severe acute respiratory syndrome (SARS).^15^ Notably, the pneumonia, ARDS, and other tissue damage in patients with COVID-19 are often associated with the cytokine storm and host immune disorders that may jointly mediate immune pathology and worsen clinical outcomes.^16,17^ Therefore, the development of immunomodulatory therapies might be beneficial to improve treatment outcomes. As such, our phase 1 trial demonstrated that the use of UC-MSCs in patients with moderate and severe COVID-19 was safe and not associated with serious adverse events, paving the way for further evaluation of the efficacy of UC-MSCs therapy for patients with moderate to severe COVID-19 in phase 2/3 trials.

Although the use of MSCs as immune therapy to treat certain diseases in humans has been generally regarded as safe,^18^ a review of MSCs therapy studies found an increased risk of fever, but no acute infusion toxicities, infections, thrombotic/embolic events, or malignancy.^19^ Our data supports previous phase 1 and phase 2 trials of the use of allogeneic bone marrow-derived MSCs for ARDS Treatment (START) to observe adverse events or toxicity.^12,13^ Furthermore, a phase 1 study of adipose tissue-derived MSCs in ARDS also found no serious adverse events related to MSCs administration.^20^ This reflects our ongoing experience of the use of UC-MSCs in patients with COVID-19. A recent report showed that a single transfusion of 1 × 10^6^ cells/kg UC-MSCs was safe in patients with critically severe COVID-19, and might improve the clinical outcome.^21^ However, that previous study did not investigate whether multiple cycles of MSCs transfusions are safe or yield more benefits for patients with COVID-19. On the basis of our previous trials for liver disease and AIDS,^22,23^ we used three cycles of UC-MSCs transfusion at a dose of 3 × 10^7^ cells/infusion. On the one hand, all the patients, whether the received UC-MSCs or not, recovered from COVID-19 with improvement of clinical symptoms, laboratory parameters, CT images of bilateral lungs, and respiratory parameters. On the other hand, the results also indicated that multiple cycles of MSCs transfusions did not aggravate the disease severity of COVID-19.

Serum IL-6 is considered a biologically relevant biomarker associated with disease progression in COVID-19, and IL-6 receptor blocking therapy using tocilizumab might help clinical improvement in patients with severe and critical COVID-19.^24,25^ Although there was no comparative analysis between the two groups because of the small number of patients, the patients in the UC-MSCs-treatment group showed a decrease of serum IL-6. Considering the multiple immune modulatory mechanisms of MSCs, a gradual decline of IL-6 level might turn out to be a biologically relevant surrogate marker of the efficacy of MSCs treatment in patients with COVID-19. However, the underlying mechanisms require further study. Of note, among the four patients with severe disease receiving UC-MSCs treatment, the one with the highest IL-6 level showed the biggest drop in the IL-6 level and improvement of oxygenation index, suggesting that UC-MSCs treatment might have the most benefit for individuals with high levels of inflammatory cytokines. This might because the inflammatory environment that is able to enhance the immunomodulatory effects of MSCs.^26^ This hypothesis needs to be confirmed by further study.

Following the results of our phase 1 trial, one of the principle investigators of our study, Dr. Fu-Sheng Wang, co-wrote guidelines to standardize stem cell treatment for COVID-19 (http://www.most.gov.cn/gnwkjdt/202003/t20200327_152617.htm), which were issued by the Chinese Ministry of Science and Technology on 27 March 2020. Accordingly, the US-FDA has approved stem cell treatment for use in seriously ill patients with COVID-19 under what is known as ‘expanded access compassionate use’. There have been recent reports of early phase studies in patients with COVID-19 from China (NCT04252118 and NCT04288102) to investigate the use of UC-MSCs in hospitalized patients with COVID-19 who were not improving despite standard therapy. A study published in Aging and Disease claimed that the MSCs therapy was safe and contributed to the recovery of all seven patients.^20^ However, the study had several limitations, such as the small number of enrolled patients and lack of randomization and controls; therefore, no conclusions on efficacy could be drawn.

In conclusion, we conducted this phase 1 trial during the early stages of the COVID-19 outbreak to determine the safety of the use of UC-MSCs to treat COVID-19. Following this phase 1 study, we have now embarked on a multi-center, phase 2/3 randomized placebo-controlled efficacy trial to assess treatment with three intravenous doses of MSCs compared with placebo, which aims to recruit 90 patients with severe COVID-19 (ClinicalTrials.gov Identifier: NCT04288102).

## Methods

### Study patients

The study was approved by the ethics committee of the Fifth Medical Center belonging to the PLA General Hospital, Beijing, China. Patients admitted to our wards were appropriately counseled, informed about the study, and written consent were obtained from each of them. The age, sex, severity of disease, co-existing diseases, the interval between symptom onset and admission, and ongoing medical treatments were recorded.

The inclusion criteria were: (1) Either male or female, aged 18–70 years old. (2) Confirmed COVID-19 cases diagnosed using reverse-transcription polymerase chain reaction (RT-PCR) (GeneoDX Co. Ltd. Shanghai, China) from nasal swab samples according to the guidelines of management of COVID-19 issued by National Health Commission of China. (3) Pneumonia was evidenced using chest radiography or CT. Exclusion criteria were: (1) Pregnancy, lactation, and those who are not pregnant but do not take effective contraceptives measures. (2) Patients with a malignant tumor, other serious systemic diseases, or psychosis. (3) Patients who are participating in other clinical trials. (4) Inability to provide informed consent or to comply with blood test requirements. (5) Co-infection of HIV, tuberculosis, influenza virus, adenovirus, and other respiratory pathogens. Moderate COVID-19 disease cases were defined as fever, respiratory symptoms, and confirmed pneumonia on CT imaging or X-ray. Severe COVID-19 cases were defined as any of the following: shortness of breath or dyspnea after activity, respiratory rate ≥30/min; oxygen saturation ≤93% at rest state; or oxygenation index (FiO2) <300 mmHg. Recovery was defined as patients who were afebrile for more than 3 days, resolution of respiratory symptoms, improvement of chest CT images, and two consecutive negative RT-PCR tests for viral RNA in respiratory tract swab samples obtained at least 24 h apart.^27^

### Study protocol

This was a very early clinical trial of UC-MSCs treatment for patients with COVID-19. There were lack of solid data and hypothesis to support calculation of the sample size in this phase I trial; therefore, 18 patients with COVID-19 admitted between 27 January to 30 March 2020 were enrolled into the study: Nine were assigned to the UC-MSCs treatment group and nine to the control group. The patients in each group were approximately matched for sex, age, and clinical characteristics.

The UC-MSCs were manufactured by Vcanbio Cell & Gene Engineering Ltd., (Tianjin, China) in a GMP facility, which had been approved by the authorities. Briefly, the Wharton’s jelly from umbilical cord tissue was diced into cubes in ~2 mm^3^, and seeded into a T75-cm^2^ tissue culture flask with MSCs culture medium. The adherent cells were digested and re-plated for expansion to master cell bank at passage 2. In the next step, cells were thawed and expended with serum-free medium. Finally the homogenous population of these cultured cells at passage 5 were collected as mentioned,^28^ and used for all experiments. The collected cells were identified to meet the minimal criteria of MSCs according to ISCT (International Society of Cell Therapy) standard.^29^ The quality and viability of these cells was reconfirmed after preparation and before each infusion for patient. MSCs are characterized by a fibroblast-like morphology, and by expression of cell surface markers including CD19, CD34, CD11b, CD45, CD73, CD105, CD90, and HLA-DR, and with potential to differentiate into osteoblasts, adipocytes, and chondroblasts (Supplementary Figs. 3 and 4). The treatment group received three cycles of intravenous infusion of allogeneic UC-MSCs (3 × 10^7^ cells each infusion) on days 0, 3, and 6. The total volume of the UC-MSCs infusion was 60 ml.

**Fig. 3:**
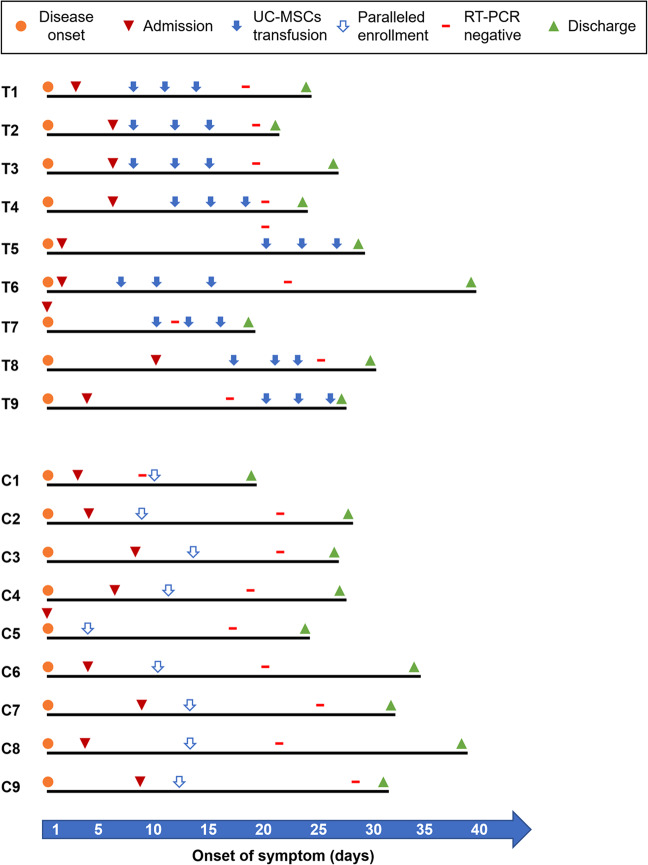
The time-axis of the enrolled patients. The key time point of each patient including disease onset, admission, UC-MSCs transfusion, paralleled enrollment, and discharge are listed

Both groups received standard care comprising recommended COVID-treatment regimens. The times of symptoms onset, admission, UC-MSCs transfusion, paralleled enrollment site, time for RT-PCR to turn negative, and discharge were documented for all 18 patients (Fig. 3). All patients continued with their routine medications.

### Safety, adverse events, and primary efficacy evaluation

All the patients were evaluated daily in our unit before their discharge. The UC-MSCs-treated patients were monitored and screened for adverse events including new symptoms and signs, and changes in body temperature, by systematic examination (including skin, eyes, and mouth), laboratory tests, and respiratory system parameters.

The clinical parameters including the duration of clinical symptoms, changes on serial chest images, the oxygenation index (PaO2/FiO2 ratio), ventilation requirements, length of hospitalization, and the interval between admission and discharge were carefully recorded (Supplementary Table 1). Laboratory tests, including CRP, liver and renal function tests (ALT and creatinine), platelets, D-dimer, and SF, were recorded in the two groups.

### Measurement of IgM and IgG antibodies against SARS-CoV-2

The titers of IgM and IgG antibodies against SARS-CoV-2 in plasma samples were tested using enzyme linked immunosorbent assay (ELISA) kits supplied by Bioscience Biological Pharmacy Enterprise (Beijing, China) according to the manufacturer’s instructions. Two patients in the control group were unwilling to have blood samples drawn for antibody detection at discharge; therefore, only seven patients were included in control group for antibody analysis.

### Plasma cytokine analysis by using aimplex kit assay

Blood samples at baseline and at day 3, 7, and 14 after UC-MSCs treatment were collected, the inflammatory cytokines (IL-6, IFN-γ, TNF-α, MCP-1, IP-10, IL-22, IL-1RA, IL-18, IL-8, and MIP-1α) were tested using flow cytometry based an Aimplex kit (Aimplex Bioscience Inc., Pomona, CA, USA) according to the manufacturer’s protocol.^30^

### Statistical analysis

The clinical and laboratory variables were summarized using descriptive statistics and confidence intervals. The *χ*² test or Fisher’s exact test was used to analyze categorical data, while the Mann–Whitney *U* test was used to compare the difference between the two groups. A *P* value of <0.05 was considered statistically significant.

## Supplementary information

Supplementary Materials

## Data Availability

All of the data generated and analyzed during this study are included in our manuscript.
