# Supplementary material for: Human umbilical cord-derived mesenchymal stem cell therapy in patients with COVID-19: a phase 1 clinical trial
Source: Signal Transduct Target Ther. 2020 Aug 27;5:172. doi: 10.1038/s41392-020-00286-5 (PMC7450163; doi:10.1038/s41392-020-00286-5)
Supplement: Supplementary file 1 — Supplementary Materials [file 41392_2020_286_MOESM1_ESM.pdf]

Supplementary Materials for

Human umbilical cord-derived mesenchymal stem cell therapy in patients with COVID-19: a phase 1 clinical trial

Fanping Meng #1, Ruonan Xu #1, Siyu Wang #1, Zhe Xu #1, Chao Zhang1, Yuanyuan Li1, Tao Yang1, Lei Shi1, Junliang Fu1, Tianjun Jiang1, Lei Huang1, Peng Zhao1, Xin Yuan1, Xing Fan1, Ji-Yuan Zhang1, Jinwen Song1, Dawei Zhang1, Yanmei Jiao1, Limin Liu1, Chunbao Zhou1, Markus Maeurer2, Alimuddin Zumla3, Ming Shi 1, Fu-Sheng Wang 1
# These authors contributed equally to this work

Prof. F-S Wang, Department of Infectious Diseases, Fifth Medical Center of Chinese PLA General Hospital, Beijing 100039, China.

Email: fswang302@163.com

Prof. M Shi, Department of Infectious Diseases, Fifth Medical Center of Chinese PLA General Hospital, Beijing 100039, China.

Email: shiming302@sina.com

This PDF file includes:

Figures. S1 to S4
Table S1

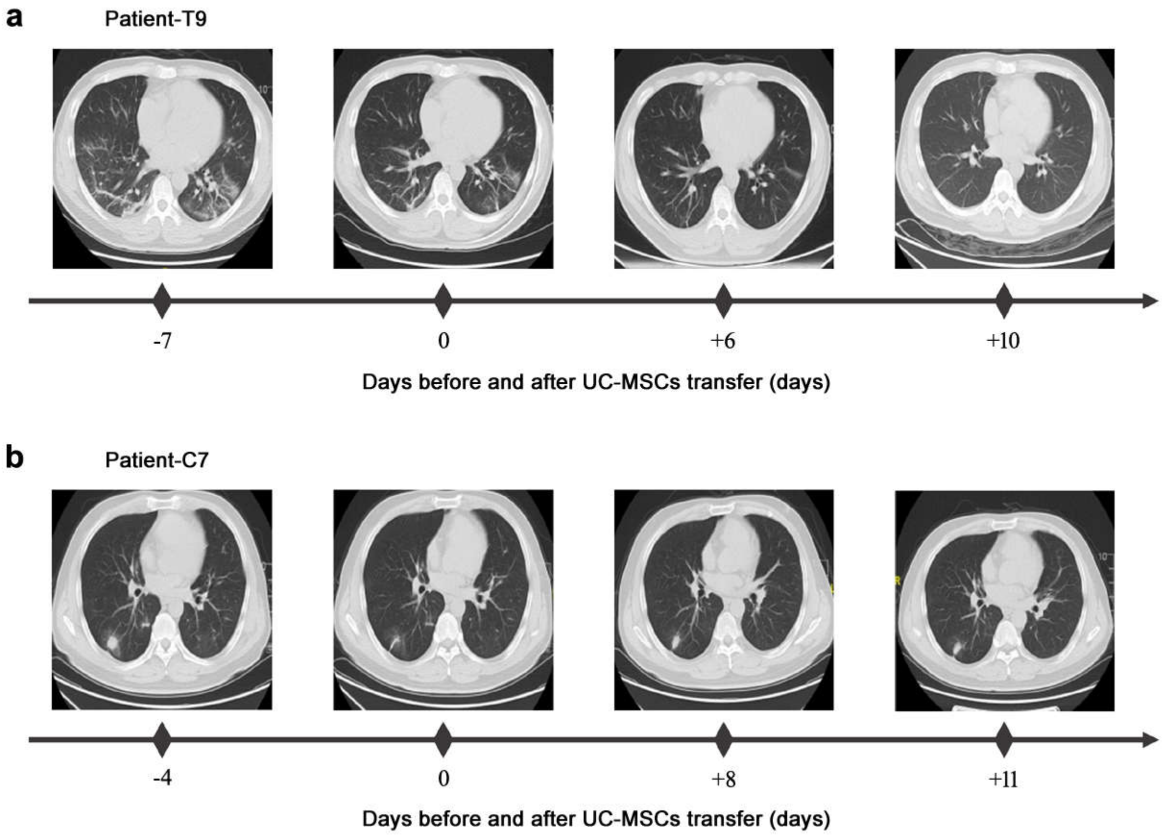

Figure. S1.

Representative chest computer tomography for the patients with COVID-19 with/without UC-MSCs transfusion.

a Representative chest CT scan images of severe patient 9 after UC-MSCs transfusion.
b Representative chest CT scan images of severe patient 7 in the control group.

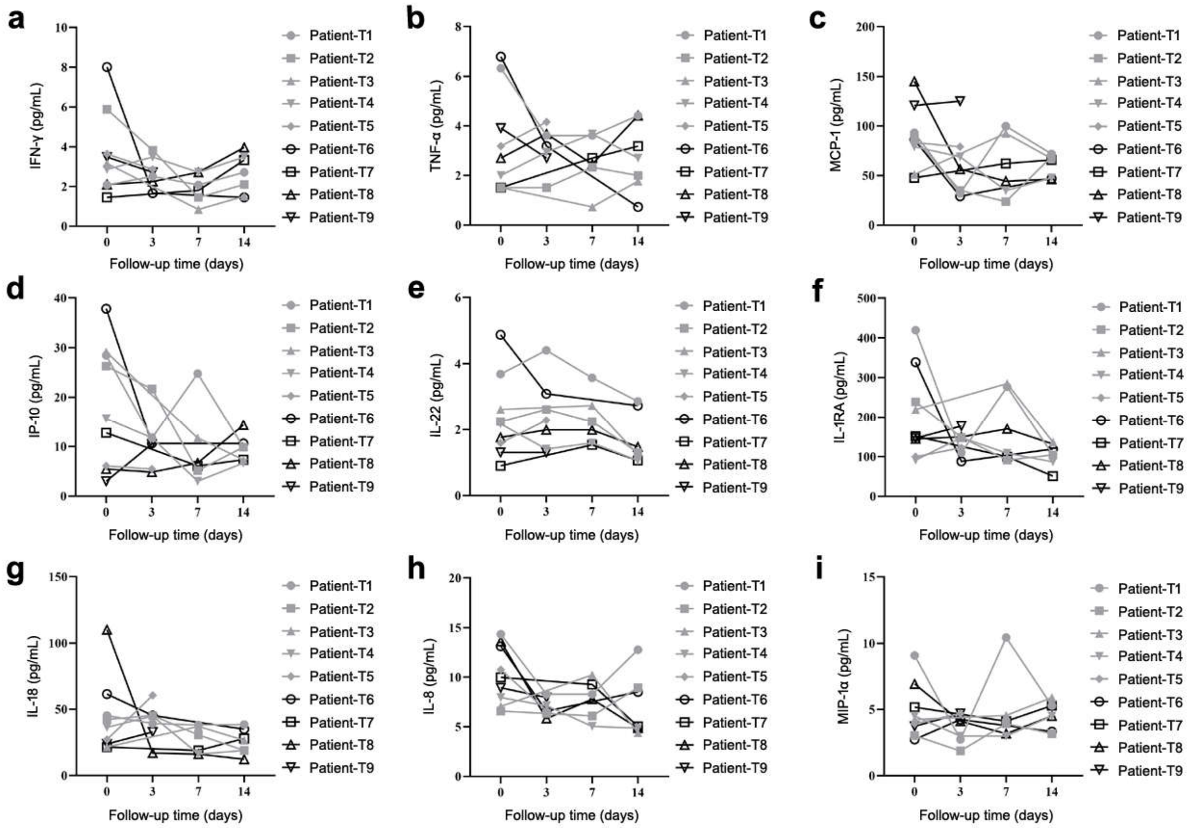

Figure. S2.

The dynamics of inflammatory cytokines in the UC-MSCs treatment group.

a-i The dynamics of plasma inflammatory cytokines within 14 days after UC-MSCs transfer are shown, including IL-6, IFN-γ, TNF-α, MCP-1, IP-10, IL-22, IL-IRA, IL-18, IL-8, and MIP-1α.

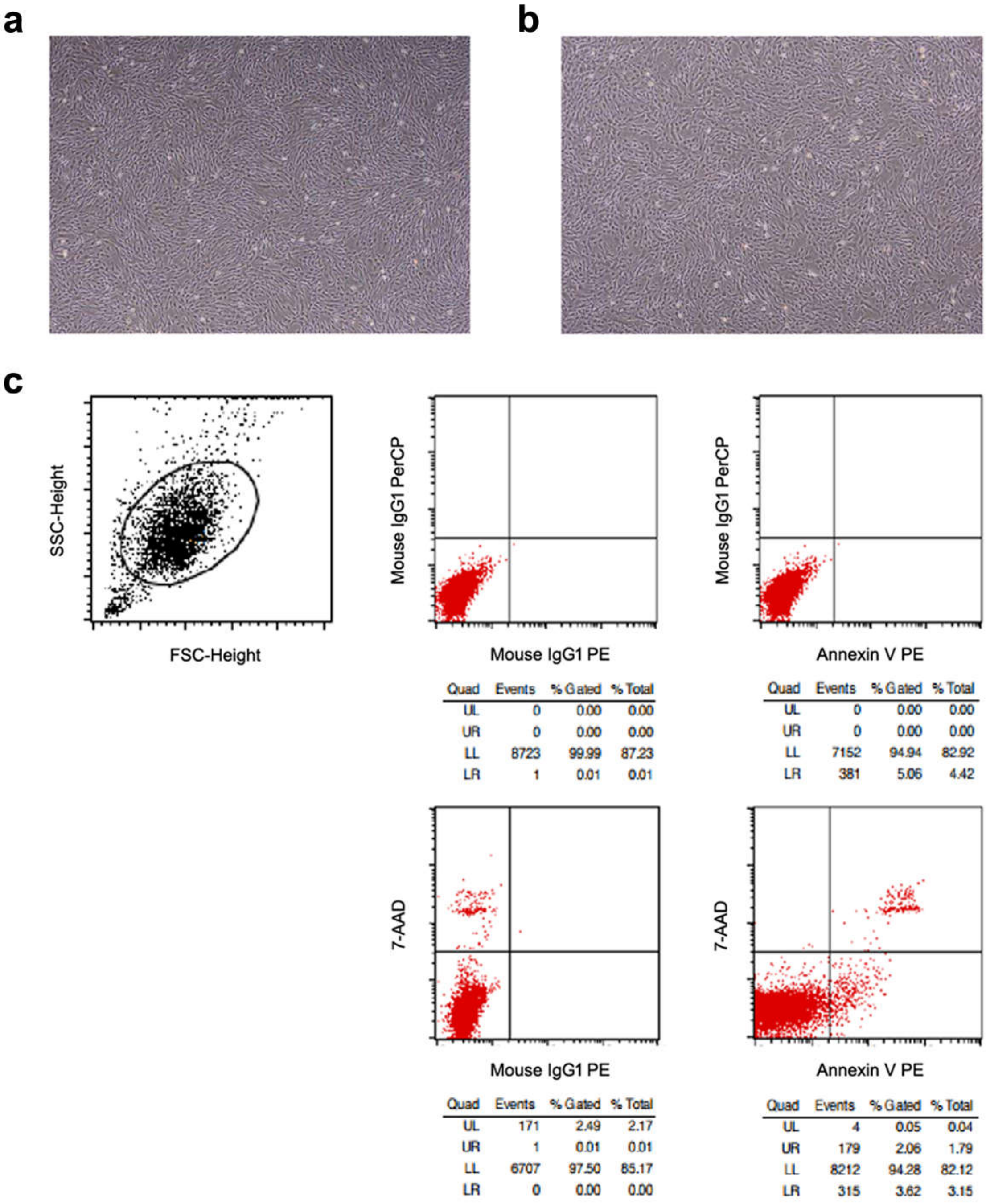

Figure. S3.

UC-MSCs morphology and viability.

a,b The UC-MSCs morphology (40 ×) and viability of working cell bank (passage 4) and final product (passage 5). **c** The apoptosis of UC-MSCs was detected using an Annexin V-/7-AAD-assay and the viability of the final product.

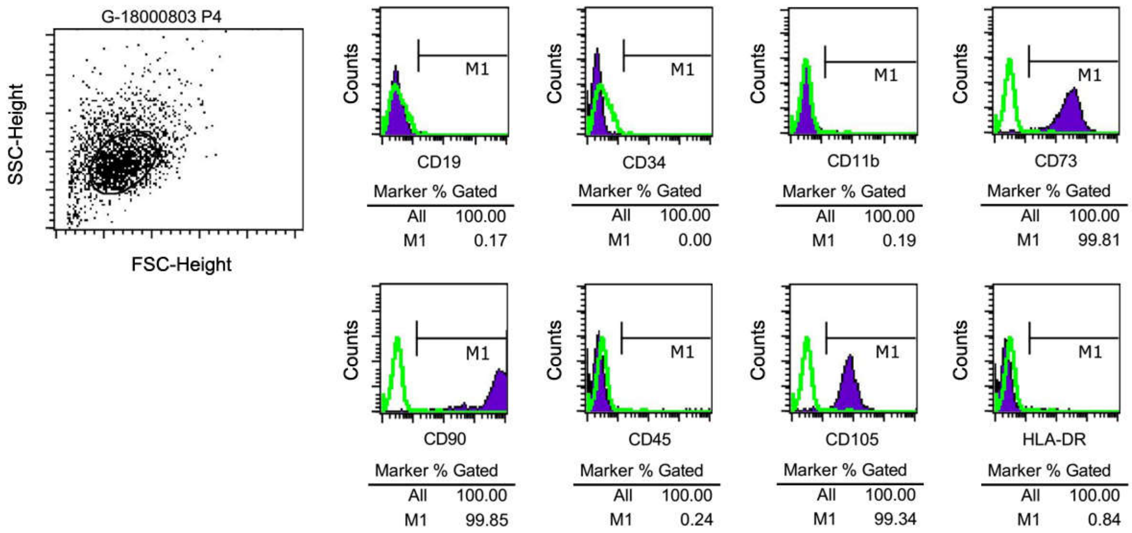

Figure. S4.

Surface markers of UC-MSCs.

The surface markers of UC-MSCs (passage 5) were analyzed using flow cytometry.

Table S1.

Details of clinical data among the patients with COVID-19 with/without UC-MSCs treatment at discharg

Table S 1. Details of clinical data among the patients with COVID-19 with/without UC-MSCs treatment at discharge.

Patient number	UC-MSCs treatment group (n=9)									Control group (n=9)								
	T1	T2	T3	T4	T5	T6	T7	T8	T9	C1	C2	C3	C4	C5	C6	C7	C8	C9
Mechanical ventilation (days)	0	0	0	0	0	1	0	0	0	0	0	0	13	0	11	0	11	5
Duration of oxygen support (days)																		
Low flow oxygen	14	0	20	0	12	21	8	12	5	0	0	7	10	16	18	22	13	1
High flow oxygen	0	0	0	0	0	14	0	1	2	0	0	0	10	0	6	2	3	20
Duration of clinical symptoms（days）																		
Fever	2	1	5	15	0	0	1	0	0	0	4	0	0	0	0	3	0	0
Fatigue	2	0	8	0	0	5	7	0	0	0	0	0	16	3	0	4	9	3
Cough	5	0	0	19	0	5	7	0	0	2	0	2	14	9	1	5	4	7
Shortness of breath (days)	0	0	0	0	0	0	3	0	0	0	0	0	14	0	2	2	9	9
Antibody at discharge (Titer of antibody, s/co)																		
Titer of IgM at discharge	66.75	4.02	6.96	32.69	9.89	59.25	9.99	31.58	24.62	/	55.56	/	512.78	19.13	88.79	3.04	76.89	187.19
Titer of IgG at discharge	20.56	4.27	34.29	2.47	4.57	32.88	19.93	44.21	1.00	/	15.69	/	19.74	21.5	121.11	13.47	97.69	122.79
Interval between admission and discharge (days)	19	15	20	17	26	37	18	20	23	16	24	19	21	23	30	23	34	22
